# Risk factors of early childhood caries among preschool children in Shanghai, China: a longitudinal study

**DOI:** 10.3389/froh.2025.1635569

**Published:** 2025-08-14

**Authors:** Yating Xu, Minyi Xu, Weihua Zhang, Xiping Feng, Jingyu Zhan, Yu Zhang, Xi Chen

**Affiliations:** ^1^ Department of Preventive Dentistry, Shanghai Ninth People’s Hospital, Shanghai Jiao Tong University School of Medicine, College of Stomatology, Shanghai Jiao Tong University, National Center for Stomatology, National Clinical Research Center for Oral Diseases, Shanghai Key Laboratory of Stomatology, Shanghai Research Institute of Stomatology, Shanghai, China; ^2^ Department of Nursing, Shanghai Pudong New Area Eye and Dental Diseases Prevention and Treatment Center, Shanghai, China

**Keywords:** early childhood caries, incidence, risk factors, longitudinal study, dental caries

## Abstract

**Introduction:**

Early childhood caries(ECC) has caused a growing public health burden worldwide, but there still remains a gap in the understanding of ECC in Shanghai, China. This study aims to investigate the current profile of ECC and related risk factors of new-onset caries among preschool children in Shanghai, China.

**Materials and methods:**

Children aged 3–5 were included in this two-year longitudinal study from Shanghai. Oral health information and related factors were collected through clinical examinations and questionnaires. Logistic and general linear regression were used to investigate the risk factors of early childhood caries. Ethical approval and informed consent form were achieved in the study.

**Results:**

A total of 192 children completed this 2-year longitudinal study with a follow-up rate of 80.0%. At follow-up, 70.8% of children developed new caries. The study revealed that children with lower mother's education level (*p* = 0.022), less father's income (*p* = 0.023), more frequent sugar intake (*p* = 0.006), and poor oral hygiene (*p* = 0.012) were more likely to develop new caries. Additionally, general linear regression demonstrated that children who did not use fluoride toothpaste (*p* = 0.006), those who consumed candy more (*p* = 0.028), and those with poor oral hygiene (*p* = 0.001) exhibited greater incremental caries.

**Conclusion:**

Lower father's income, lower mother's education level, frequent sugar consumption, non-use of fluoridated toothpaste, and poor oral hygiene status emerged as significant independent risk factors for ECC.

## 1 Introduction

Dental caries, one of the most prevalent chronic diseases, have become a major public health burden worldwide (1, 2). Due to changes in dietary patterns, lower mineralization of deciduous teeth compared to permanent teeth, and weaker awareness of oral self-care among children, the prevalence of dental caries in preschool children remains persistently high (3). Almost half of preschool children globally are affected by dental caries (4). In China, the mean decayed, missing, or filled primary teeth index (dmft) among 5-year-old children has shown a significant increase over the past decade, rising from 3.5 in 2005 to 4.2 in 2015 (5, 6). During the same period, caries prevalence in this age group increased from 66.0% to 71.9%, reflecting a 5.9-percentage-point absolute increase (5, 6). Moreover, Dental caries in preschool children exhibit early onset, high prevalence, with a continuous upward trend, and low treatment rates.

Early childhood caries (ECC)is a clinically aggressive form of dental decay that typically appears shortly after the eruption of teeth. It primarily affects smooth enamel surfaces, progresses rapidly, and can lead to significant long-term consequences for dental health (7). In the early stages, children with dental caries often do not show obvious symptoms. If timely intervention is not provided, these children may experience pain and infection, which can escalate into more serious complications such as difficulties with feeding, sleep deprivation, chronic systemic infections, growth retardation, and psychological issues (7, 8). There is currently a significant gap between the preventive measures for ECC in China and those in developed countries such as Japan and the United States, indicating that China still faces substantial challenges in ECC prevention (8, 9). Significant variations in ECC epidemiology across different countries and regions necessitate investigating its risk factors and developing prevention strategies specifically tailored to China's unique context (9).

ECC is a multifactorial public health issue influenced by diet, oral hygiene, socioeconomic factors, and guardians’ attitudes, among others (10–12). Since there have been few longitudinal studies exploring the risk factors of ECC in China, the majority of research has concentrated on the prevalence and incidence of ECC. Wang et al. established a cohort of preschool children aged 3–4 years in Wenzhou, China, reporting a high incidence and prevalence of ECC (13). Similarly, Zhou et al. examined the early life influences in an 8-month-old cohort in Guangzhou, China (14). These gaps highlight an urgent need for longitudinal studies identifying ECC risk factors across key developmental stages, particularly 3–5 years old, in China.

Our previous study based on a one-year follow-up study revealed that residing in suburban areas, non-use of fluoride toothpaste, and frequent consumption of sugary snacks are significant risk factors for the onset of caries in preschool children (15). Building on these findings, this study conducted a two-year investigation among preschool children in Shanghai, China, further exploring risk factors for newly emerged ECC, identifying patterns of disease development and susceptible populations, and controlling the onset and progression of dental caries. Ultimately, the goal is to provide evidence and guidance for prevention strategies to reduce the incidence of ECC.

## 2 Subjects and methods

### 2.1 Sampling strategy and inclusion criteria

In this study, the sample size was calculated based on the data of the fourth national oral health survey, in which the prevalence of dental caries was 71.9%. To meet the follow-up rate of 80%, the final sample size was 160. A multistage cluster sampling was used in this study. Six kindergartens were randomly selected in Shanghai. Subsequently, two classes per preschool grade were randomly chosen using cluster sampling, with 20 students meeting the inclusion criteria enrolled from each class.

Participants were required to meet the following inclusion criteria at baseline: They must provide signed informed consent, be residents of Shanghai, be aged between 3 and 5 years old, and demonstrate good compliance. The exclusion criteria included children who were unable to cooperate during dental examinations, guardians who did not complete questionnaires, or children with systemic diseases.

### 2.2 Clinical examination and questionnaire survey

#### 2.2.1 Clinical examination

All preschoolers underwent oral examinations by pediatric dentists under good illumination. Dental caries was diagnosed by visually inspecting and probing the teeth. To improve visibility, the tooth surfaces were dried with cotton rolls. Examinations were performed using a headlamp, a disposable dental mirror, and a WHO-CPI probe. Caries assessment followed the World Health Organization (WHO) standards from the Oral Health Surveys: Basic Methods (5th Edition) (14). Caries status was recorded using the decayed, missing, filled teeth for primary teeth index (dmft) and the decayed, missing, filled surfaces index (dmfs). Dental plaque coverage on all tooth surfaces was assessed, and the number of specific teeth with plaque presence was recorded, documenting this as the Visible Plaque Index (VPI). Oral hygiene status is evaluated using the VPI, with scores ranging from 0 to 8 indicating good oral hygiene, while scores from 9 to 20 indicate poor oral hygiene.

#### 2.2.2 Indicator definition

Incremental caries per child: For preschool children in the study, new caries was defined as the emergence of decay in previously unaffected tooth (surfaces) positions. Δdmft and Δdmfs were calculated as:

-Δdmft = dmft at 2-year follow-up—dmft at baseline;

-Δdmfs = dmfs at 2-year follow-up−dmfs at baseline.

New caries incidence rate: The proportion of children developing new caries during follow-up was calculated as:

-New caries incidence rate = (Number of children with Δdmft ≥1/total examined participants) × 100%.

#### 2.2.3 Quality control

Before the formal data collection, examiners took part in a pilot study that was supervised by certified examiners from the Fourth National Oral Health Survey. During the clinical examinations, 5% of participants were randomly selected for re-examination to evaluate intra-examiner reliability, achieving a Kappa value greater than 0.8. Additionally, inter-examiner reliability was validated through consistency checks, which also showed a Kappa value greater than 0.8.

#### 2.2.4 Questionnaire survey

The questionnaire was created based on reference to the Fourth National Oral Health Survey in the Mainland of China, the WHO Children's Oral Health Questionnaire, and the 2009 China Health and Nutrition Survey Questionnaire, incorporating risk factors for ECC. It covered the following domains: children's demographic information, family socioeconomic background, general health status, feeding practices, dietary habits, oral hygiene practices, parental attitudes towards oral health knowledge, and healthcare-seeking behaviors.

### 2.3 Data analysis

Statistical analysis was performed using IBM SPSS Statistics 24.0 software. Initially, descriptive methods were applied to analyze the general characteristics and dental caries status of the study participants. Independent samples *t*-tests, Chi-square tests, and analysis of variance (ANOVA) were utilized for univariate analysis of caries status to screen risk factors associated with new caries incidence rate and incremental caries per child. Logistic regression and general linear regression analyses were employed to develop predictive models for new-onset caries in primary teeth, incorporating variables with *p* < 0.2 into the regression equations. The significance level was set at *α*=0.05, and differences were considered statistically significant at *p* < 0.05.

## 3 Results

### 3.1 Study participants and ECC characteristics

A total of 240 preschool children (mean age: 4.2 ± 0.3 years old, range:3.67–4.65 years old) participated in this survey at baseline. Out of these, 192 children completed the 2-year follow-up, achieving a follow-up rate of 80%. The loss to follow-up, which involved 48 children, was primarily due to school transfers or absences. No statistically significant differences were observed between the lost-to-follow-up group and the retained cohort in terms of gender, parental education level, or residential location (*p* > 0.05).

The 240 children exhibited a caries prevalence of 58.4%,a mean dmft score of 3.1 ± 4.2 and a mean dmfs score of 5.5 ± 9.6 at baseline. After 2 years of follow-up, the 192 children showed a caries prevalence of 76.0% and a mean dmft of 5.4 ± 5.0. Compared to the baseline, the overall new caries incidence rate was 70.8%, with an incremental caries per child (Δdmft) of 2.2 ± 2.2 and an incremental caries surfaces per child (Δdmfs) of 8.7 ± 9.0. As described in Table 1, neither dmft, dmfs, nor caries prevalence at baseline or the 2-year follow-up was associated with gender (*p* > 0.05). Children residing in suburban areas exhibited significantly higher caries prevalence, dmft scores, and dmfs scores compared to their urban counterparts at both baseline and the 2-year follow-up (*p* < 0.05).

**Table 1:** Sample distribution and dental caries experience of children in the study.

Variable	Baseline				2-year follow-up					
	Prevalence of dental caries (%)	*p* ^#^	Dmft (mean ± SD)	*p* ^‡^	Prevalence of dental caries (%)	*p* ^#^	Dmft (mean ± SD)	*p* ^‡^	Dmfs (mean ± SD)	*p* ^‡^
Gender		0.334		0.964		0.746		0.690		0.721
Male	55.4		3.0 ± 4.3		75.0		5.3 ± 5.1		16.6 ± 1.73	
Female	61.5		3.1 ± 4.0		77.0		5.4 ± 4.9		14.3 ± 16.6	
Location		0.003		0.003		0.006		< 0.001		< 0.001
Suburb	62.9		3.6 ± 4.5		81.3		6.1 ± 5.2		16.8 ± 18.0	
Urban	47.9		1.9 ± 2.9		62.3		3.5 ± 3.8		7.5 ± 9.1	

dmft, decayed, missing, and filled teeth; dmfs, decayed, missing, and filled surfaces.

^‡^ *p* value derived from *t*-tests for independent samples.

^#^ *p* value derived from Chi-square tests.

### 3.2 Relationships between ECC and questionnaire results

This study revealed that the education levels of parents, paternal income, and children's residential location were associated with incremental caries tooth and caries surfaces per child and new caries incidence rate (Table 2). As paternal education level increased, children exhibited a decreasing trend in new caries incidence rate (*p* = 0.015), as well as Δdmft(*p* = 0.031), and Δdmfs (*p* = 0.010). Similarly, higher maternal education level was linked to a lower new caries incidence rate (*p* = 0.006), reductions in Δdmft (*p* = 0.018), and Δdmfs (*p* = 0.003). Higher paternal income was also linked to a decreased new caries incidence rate (*p* = 0.002) and Δdmfs (*p* = 0.006). Additionally, the residential location had a significant impact on caries outcomes, with children living in suburban areas showing a higher incidence of new caries and greater incremental changes in the dmfs and dmft indices compared to those in urban areas over a 2-year follow-up period (*p* < 0.05).

**Table 2:** Association between new caries incidence indicators and socioeconomic status.

Factors	*n*	New caries incidence rate (%)	*p*	Δdmft (mean ± SD)	*p*	Δdmfs (mean ± SD)	*p*
Gender			0.958^#^		0.505^‡^		0.386^‡^
Male	92	70.7		2.1 ± 2.0		8.1 ± 8.2	
Female	100	71.0		2.3 ± 2.3		9.3 ± 9.7	
Location			0.002^#^		0.021^‡^		< 0.001^‡^
Suburb	139	77.0		2.5 ± 2.2		10.2 ± 9.6	
Urban	53	54.7		1.6 ± 2.0		4.8 ± 5.6	
Mother's education level^a^			0.006^#^		0.018^‡^		0.003^‡^
College or below	92	80.4		2.6 ± 2.2		10.8 ± 9.0	
Bachelor or higher	95	62.1		1.9 ± 2.1		6.9 ± 8.7	
Father's education level^a^			0.015^#^		0.031^‡^		0.010^‡^
College or below	88	79.5		2.6 ± 2.3		10.6 ± 9.2	
Bachelor or higher	98	63.3		1.9 ± 2.0		7.2 ± 8.7	
Father's monthly income^a^			0.002^#^		0.120^§^		0.006^§^
≤ 6,000 CNY	56	85.7		2.6 ± 2.1		11.2 ± 10.0	
6,000–12,000 CNY	63	69.8		2.4 ± 2.2		8.7 ± 9.0	
> 12,000 CNY	58	55.2		1.8 ± 2.2		5.9 ± 7.2	

^a^ Missing values are present for this item.

^‡^ *p* value derived from *t*-tests for independent samples.

^§^ *p* value derived from analysis of variance (ANOVA).

^#^ *p* value derived from Chi-square tests.

Oral health-related behaviors, including feeding practices, oral hygiene habits, and dietary patterns, were analyzed for their associations with new-onset caries (Table 3). Children with poor oral hygiene demonstrated significantly higher risks across all caries metrics (*p* < 0.05). Interestingly, recent dental visits within 6 months showed elevated caries risk (*p* = 0.008), which may reflect care-seeking behavior triggered by symptoms of dental issues. Additionally, non-use of fluoride toothpaste (*p* = 0.006) and weekly candy consumption (*p* = 0.004) independently increased Δdmft. However, infant feeding methods, duration of breastfeeding, toothbrushing frequency, consumption of biscuits/cakes, fruits, soft drinks, honey/jam, or snacks, as well as the frequency of snacks between meals were not significantly associated with Δdmft, Δdmfs or new caries incidence rate (Supplementary Table S1, *p* > 0.05).

**Table 3:** Association between new caries incidence indicators and oral health-related behaviors.

Factors	*n*	New caries incidence rate (%)	*p* ^#^	Δdmft (mean ± SD)	*p* ^‡^	Δdmfs (mean ± SD)	*p* ^‡^
Oral hygiene			< 0.001		0.003		< 0.001
Good	93	58.1		1.80 ± 2.20		6.10 ± 7.10	
Poor	99	82.8		2.70 ± 2.20		11.20 ± 9.90	
Fluoride toothpaste use^a^			0.063		0.006		0.205
Yes	89	64.0		1.76 ± 2.03		7.89 ± 9.25	
No	94	76.6		2.66 ± 2.28		9.61 ± 9.04	
Dental check-up in the past 6 months^a^			0.008		0.511		0.120
No or unsure	115	63.5		2.18 ± 2.21		8.22 ± 8.85	
Yes	75	81.3		2.44 ± 2.45		10.71 ± 9.54	
Frequency of consuming candy^a^			0.054		0.004		0.277
Once weekly or less	118	66.1		1.91 ± 2.03		8.23 ± 8.99	
Twice weekly or more	68	79.4		2.87 ± 2.36		9.74 ± 9.24	

^a^ Missing values are present for this item.

^‡^ *p* value derived from *t*-tests for independent samples.

^#^ *p* value derived from Chi-square tests.

### 3.3 Multiple regression analysis of ECC risk factors

Independent variables with *p* < 0.2 in the univariate analysis of new caries incidence rate were incorporated into a multivariate logistic stepwise backward regression model. As indicated in Table 4, the results demonstrated that children whose mothers with an education level below college (*p* = 0.022), fathers with a monthly income below 6,000 CNY (*p* = 0.023), higher weekly frequency of sugary snack consumption (*p* = 0.006), or poor oral hygiene status (*p* = 0.012) exhibited a significantly higher new caries incidence rate (*p* = 0.009).

**Table 4:** Logistic regression results of risk factors for new caries incidence in deciduous teeth.

Factors	OR	95% CI	*p*
Mother's education level			0.022
College or below^a^			
Bachelor or higher	0.387	0.173–0.870	
Father's monthly income			0.023
≤ 6,000 CNY^a^			
6,000–12,000 CNY	0.390	0.138–1.014	0.076
> 12,000 CNY	0.327	0.110–0.974	0.045
Frequency of consuming candy			0.006
Once weekly or less^a^			
Twice weekly or more	3.311	1.408–7.778	
Oral hygiene			0.012
Good^a^			
Poor	2.833	1.254–6.399	
Intercept	3.915		0.009

CI, confidence interval.

^a^ Reference group.

For Δdmft, independent variables with *p* < 0.2 in the univariate analysis were selected and included in a general linear regression model (Table 5). The regression analysis revealed that children who did not use fluoride toothpaste (*p* = 0.006) or consumed sugary snacks more than once weekly (*p* = 0.028) had significantly higher incremental caries per child (*p* < 0.001). Additionally, as presented in Table 6, consistent variable selection criteria were applied, revealing that children with poor oral hygiene status (*p* = 0.001) had significantly higher Δdmfs (*p* = 0.021).

**Table 5:** General linear regression results of risk factors for mean Δdmft in deciduous teeth.

Factors	Parameter estimates	95% CI	*p*
Fluoride toothpaste use			0.006
Yes	−0.911	−1.559 to −0.262	
No^a^			
Frequency of consuming candy			0.028
Once weekly or less	−0.914	−1.580 to −0.247	
Twice weekly or more^a^			
Intercept	2.387	1.472–3.302	< 0.001

^a^ Reference group.

**Table 6:** General linear regression results of risk factors for mean Δdmfs in deciduous teeth.

Factors	Parameter estimates	95% CI	*p*
VPI	0.457	0.180–0.734	0.001
Intercept	4.512	0.688–8.335	0.021

VPI, visible plaque index.

## 4 Discussion

This study revealed a caries prevalence of 76.0% among senior-class preschool children in Shanghai, surpassing the 71.9% prevalence rate reported for 5-year-olds in the Fourth National Oral Health Survey in the Mainland of China (5). The higher prevalence may be that the subjects we included in this study were more from the suburbs of Shanghai. Moreover, the overall new caries incidence rate was 70.8% in this two-year follow-up study. The findings demonstrated a potentially more severe prevalence of dental caries among preschool children in Shanghai suburbs compared to previous estimates or national averages, indicating the urgent need for heightened awareness and targeted interventions.

The study investigated the association between socioeconomic backgrounds and ECC. Father's monthly income and education level, and mother's education level were inversely correlated with new caries incidence rates. Children from suburban areas exhibited significantly higher new caries incidence rates compared to urban children. Parents play a significant role in children's oral health behaviors and habit formation (16, 17). Previous studies have also reported significant relationships between minders’ attitudes, knowledge, oral health status, oral health habits and ECC development (18–20). Du et al. demonstrated that ECC prevalence was higher in children from low-income families. The main factors contributing to inequality were average household income, parents’ educational level, and living areas (21). Furthermore, the limited access to nutrient-dense foods among low-income families results in dependence on high-energy, low-cost, sugar-laden diets, which serve as a critical etiological factor in caries pathogenesis (22). These findings emphasized the importance of enhancing parents’ awareness of oral health care, which is crucial for fostering better oral health outcomes and laying a solid foundation for children's long-term dental well-being. Given that low paternal income and low parental education levels are well-established risk factors for ECC, leveraging external support to enhance parental health literacy is crucial. Furthermore, where feasible, governmental and community programs should supply oral health kits, like toothbrushes and dental floss, and offer subsidized dental care to individuals from low-income households, thereby alleviating financial barriers to oral healthcare access.

This study found that residential location significantly influenced new caries incidence, with children living in suburban areas exhibiting a higher rate than those in urban areas (*p* = 0.002), which was consistent with previous findings (15, 23, 24). This may be due to systemic health inequities of suburban areas characterized by poverty, lower education, and fewer oral healthcare providers. Interestingly, in the multiple regression model, location failed to demonstrate statistical significance as a predictor variable, which may be the result of the uneven distribution of participants across urban and suburban groups. This study suggests that suburban children need to pay more attention to oral health, which requires strengthening preventive measures and increasing access to dental healthcare services. Concurrently, key stakeholder groups including preschool teachers, community health workers, and primary care providers should be actively engaged to facilitate timely, community-based interventions for ECC. Recent studies suggest that insufficient knowledge and training in children's oral health and ECC management may exist even among pediatric specialists (25). Governments need to strengthen the capacity of primary dental care systems in suburban areas. This includes training community healthcare providers to conduct ECC risk screening, deliver oral health education, and establish streamlined referral pathways.

While dental visits are typically considered protective, the study observed that recent dental visits within 6 months showed elevated caries risk (*p* = 0.008), reflecting care-seeking behavior often triggered by symptoms of dental issues. Our findings align with the Fourth National Oral Health Epidemiological Survey of China, which revealed that 50.6% of preschoolers (3–5 years old)sought dental care for treatment purposes within 12 months, vs. merely 11.6% for preventive reasons (5). Such patterns underscore an urgent need for public education on routine check-ups and a shift from symptom-driven to prevention-oriented oral healthcare.

The study further confirmed that frequent sugar consumption strongly correlated with new caries development, aligning with our earlier 1-year follow-up results (15). In recent decades, dietary habits have shifted, including the popularity of energy-dense, low-nutrient-dense foods, which are often characterized by a high content of added sugar (26). Carbohydrates play a critical role in the caries development, acting as essential metabolic substrates and promoting the growth of cariogenic bacteria (23, 27). The WHO made a conditional recommendation for a further reduction of the intake of free sugars to <5% of total energy intake (28). As reported, restricting sugary food intake as well as avoiding unhealthy snacking habits are effective strategies for caries prevention (29). Limiting children's sugar intake should extend beyond the home to kindergarten settings, where concerted efforts are needed to reduce sugar-sweetened snacks and beverages while increasing the availability of healthier alternatives. For governments, multi-pronged environmental interventions, such as promoting healthier beverages in supermarkets, restricting the availability of sugar-sweetened beverages (SSB) in schools, and implementing fiscal measures, could reduce SSB consumption (30).

Additionally, consistent with our previous study, both the 1-year follow-up and current 2-year follow-up results demonstrated that fluoride toothpaste use effectively reduced new caries incidence, corroborating global evidence (15, 31). Fluoride enhances tooth remineralization and inhibits demineralization (32). Proper use of fluoride provides significant lifelong benefits for oral health. Topical fluoride therapies effectively reduce the incidence of ECC, but it is worth noting that children's home-use fluoride products should be used in smaller doses and more frequently (33). To avoid the side effects of fluoride, guidelines suggest using a rice-grain-sized amount for children under 2 years old and a pea-sized ease for those aged 3–6 years (34). Fluoride varnish demonstrates substantial caries-preventive efficacy in both permanent and primary dentition (35). Health education initiatives should advocate for use of fluoride toothpaste, while fluoride varnish applications ought to be integrated into routine pediatric healthcare protocols.

The VPI assessment revealed that poor oral hygiene status significantly increased Δdmft and Δdmfs, and overall caries incidence. This highlights the critical role of oral hygiene maintenance in preventing caries. Poor brushing habits is significantly related to caries development (36). Given preschool children's limited cognitive abilities and weak self-care awareness, promoting parents to help their children brush their teeth is the essential method to improve tooth brushing in children and to prevent childhood dental caries. Instead of directly instructing preschool children about oral health, multiple-level dental knowledge instruction of guardians can be more effective to prevent ECC. Healthcare professionals need to pay more attention in guiding parents to improve children's brushing practices, including proper technique, adequate duration, and a twice-daily brushing routine (37). Community-level interventions should incorporate not only regularly scheduled workshops delivering step-by-step oral hygiene protocols, but also scalable digital tools (38). Platforms like Douyin/TikTok have already proven instrumental in disseminating chronic disease education offering a strategic channel to amplify ECC prevention messaging (39).

There are some limitations of this study. Firstly, the generalizability of the findings is limited due to its focus on a single city. These samples were exclusively drawn from Shanghai, an economically advanced coastal city in eastern China. Given its unique socioeconomic profile and substantial regional disparities, the findings may not represent China's national average status of ECC. Secondly, the assessment of oral hygiene knowledge of caregivers is incomplete, such as the lack of knowledge about caries prevention. Future research should balance urban and suburban ratios, and improve questionnaires to completely assess caregivers’ knowledge and behaviors.

## 5 Conclusions

The present study provides new insights into ECC-related risk factors in Shanghai. Several factors contribute to ECC disparities, including low father's income, mother's education below high school, high sugar consumption, non-use of fluoridated toothpaste, and poor oral hygiene. There is an urgent need to enhance parents’ awareness of oral health, particularly among caregivers with limited education. Children's oral health would benefit from reducing sugar intake, promoting fluoride use, and establishing good oral hygiene habits. Effective ECC prevention in China requires coordinated actions across multiple levels, from individuals and families to schools, communities, and government, tailored to local realities.

## Data Availability

The raw data supporting the conclusions of this article will be made available by the authors, without undue reservation.
