# Supplementary material for: Risk factors of early childhood caries among preschool children in Shanghai, China: a longitudinal study
Source: Front Oral Health. 2025 Aug 14;6:1635569. doi: 10.3389/froh.2025.1635569 (PMC12391158; doi:10.3389/froh.2025.1635569)
Supplement: Supplementary file 1 [file Table1.docx]

**Risk factors of early childhood caries among preschool children in Shanghai, China:**

**A longitudinal study**

**Yating Xu^1,†^, Minyi Xu^1,†^, Weihua Zhang ^2,†^, Xiping Feng^1^, Jingyu Zhan^1,^ *,Yu Zhang^1,^ *, Xi Chen^1,^***

^1^ Department of Preventive Dentistry, Shanghai Ninth People’s Hospital, Shanghai Jiao Tong University School of Medicine; College of Stomatology, Shanghai Jiao Tong University; National Center for Stomatology; National Clinical Research Center for Oral Diseases; Shanghai Key Laboratory of Stomatology; Shanghai Research Institute of Stomatology, Shanghai, China.

^2^ Department of Nursing, Shanghai Pudong New Area Eye and Dental Diseases Prevention and Treatment Center, Shanghai, China

† These authors contributed equally to this study and share first authorship.

*** Corresponding author**

Xi Chen ([chenxi9h@126.com](mailto:chenxi9h@126.com))

Department of Preventive Dentistry, Shanghai Ninth People’s Hospital, Shanghai Jiao Tong University School of Medicine, 639 Zhizaoju Road, Shanghai, PR China; Tel: +86-021-53315327.

Yu Zhang (zy24ymjulia@163.com)

Department of Preventive Dentistry, Shanghai Ninth People’s Hospital, Shanghai Jiao Tong University School of Medicine, 639 Zhizaoju Road, Shanghai, PR China; Tel: +86-021-53315327.

Jingyu Zhan (zhan0shuiya@163.com)

Department of Preventive Dentistry, Shanghai Ninth People’s Hospital, Shanghai Jiao Tong University School of Medicine, 639 Zhizaoju Road, Shanghai, PR China; Tel: +86-021-53315327.

**Table S1 Association between new caries incidence indicators and oral health-related behaviors.**

| Factors | *n* | New Caries Incidence Rate  (%) | *p* | Δdmft  (mean ± SD) | *p* | Δdmfs  (mean ± SD) | *p* |
| --- | --- | --- | --- | --- | --- | --- | --- |
| **Feeding method within 4 months** |  |  | 0.258^#^ |  | 0.277^§^ |  | 0.520^§^ |
| Breastfeeding | 38 | 81.6 |  | 2.34 ± 2.04 |  | 10.20 ± 7.45 |  |
| Mixed feeding | 65 | 69.2 |  | 2.52 ± 2.40 |  | 8.45 ± 9.67 |  |
| Formula feeding | 89 | 67.4 |  | 1.97 ± 2.06 |  | 8.28 ± 9.09 |  |
| **Duration of breastfeeding^†^** |  |  | 0.299^#^ |  | 0.929^§^ |  | 0.809^§^ |
| < 6 months | 86 | 75.6 |  | 2.26 ± 2.07 |  | 8.81 ± 8.61 |  |
| 6-12 months | 67 | 64.2 |  | 2.16 ± 2.35 |  | 8.97 ± 10.10 |  |
| > 12 months | 29 | 72.4 |  | 2.34 ± 2.27 |  | 7.69 ± 8.45 |  |
| **Maternal oral health^†^** |  |  | 0.141^#^ |  | 0.755^‡^ |  | 0.463^‡^ |
| Good  Poor | 178  11 | 70.2  90.9 |  | 2.24 ± 2.18  2.45 ± 2.42 |  | 8.66 ± 9.13  10.72 ± 7.25 |  |
| **Frequency of tooth brushing^†^** |  |  | 0.420^#^ |  | 0.833^‡^ |  | 0.331^‡^ |
| ≤ 1 time/day | 98 | 73.5 |  | 2.27 ± 2.13 |  | 9.35 ± 9.31 |  |
| > 1 time/day | 91 | 68.1 |  | 2.20 ± 2.26 |  | 8.07 ± 8.72 |  |
| **Frequency of consuming cookie/cake^†^** |  |  | 0.118^#^ |  | 0.332^‡^ |  | 0.583^‡^ |
| ≤ 1 time/week | 77 | 64.9 |  | 2.06 ± 2.27 |  | 8.35 ± 9.02 |  |
| > 1 time/week | 110 | 75.5 |  | 2.38 ± 2.13 |  | 9.19 ± 9.10 |  |
| **Frequency of consuming fruit^†^** |  |  | 0.078^#^ |  | 0.893^‡^ |  | 0.912^‡^ |
| ≤ 1 time/week | 13 | 92.3 |  | 2.15 ± 1.52 |  | 8.46 ± 6.41 |  |
| > 1 time/week | 176 | 69.3 |  | 2.24 ± 2.23 |  | 8.75 ± 9.21 |  |
| **Frequency of**  **consuming soft drink^†^** |  |  | 0.947^#^ |  | 0.887^‡^ |  | 0.462^‡^ |
| ≤ 1 time/week | 161 | 70.8 |  | 2.24 ± 2.20 |  | 8.53 ± 9.11 |  |
| > 1 time/week | 28 | 71.4 |  | 2.18 ± 2.14 |  | 9.89 ± 9.63 |  |
| **Frequency of**  **consuming jam/honey^†^** |  |  | 0.589^#^ |  | 0.214^‡^ |  | 0.744^‡^ |
| i≤ 1 time/week | 174 | 71.3 |  | 2.16 ± 2.13 |  | 8.71 ± 9.17 |  |
| > 1 time/week | 11 | 63.6 |  | 3.00 ± 2.90 |  | 9.64 ± 8.19 |  |
| **Frequency of snacks between three meals^†^** |  |  | 0.784^#^ |  | 0.429^‡^ |  | 0.694^‡^ |
| rarely not | 8 | 75.0 |  | 1.63 ± 1.60 |  | 7.50 ± 6.55 |  |
| > 1 time/day | 183 | 70.5 |  | 2.25 ± 2.21 |  | 8.79 ± 9.11 |  |

Note: †Missing values are present for this item.

^#^ *p* value derived from Chi-square tests.

^‡^ *p* value derived from t-tests for independent samples.

^§^ *p* value derived from analysis of variance (ANOVA).
